# Occupational toxic encephalopathy due to 1,2-dichloroethane exposure: a case series

**DOI:** 10.3389/fphar.2025.1542156

**Published:** 2025-09-11

**Authors:** Tongyao Li, Wensi Hu, Xia Rong, Ping Yang, Yu Du, Songbai He, Haibo Tang, Linna Luo, Lin Shi, Junzhao Liu

**Affiliations:** ^1^ Department of Critical Care and Emergency Medicine, West China School of Public Health, West China Fourth Hospital, Sichuan University, Chengdu, Sichuan, China; ^2^ Health Emergency Management Research Center, China-PUMC C.C. Chen Institute of Health, West China School of Public Health and West China Fourth Hospital, Sichuan University, Chengdu, Sichuan, China

**Keywords:** toxic encephalopathy, 1,2-dichloroethane, poisoning, occupational exposure, magnetic resonance imaging

## Abstract

1,2-Dichloroethane (1,2-DCE) is a commonly used organic solvent in industrial settings. In pharmacokinetic studies using electrochemical techniques, it is widely used as an organic solvent for dissolving drugs and forms a water/1,2-DCE interface. The 1,2-DCE exposure caused by inadequate protection poses a risk of toxicity via inhalation or dermal contact. This case series documents five instances of poisoning resulting from occupational exposure to industrial products containing 1,2-DCE. 1,2-DCE can induce neurological damage, particularly affecting the central nervous system, manifesting as toxic encephalopathy. Clinical manifestations encompass headache, limb convulsions, and coma, often accompanied by increased intracranial pressure. Magnetic resonance imaging aids in the early detection of toxic encephalopathy by revealing extensive cerebral edema and diffuse, symmetrical abnormalities in signal intensity within the bilateral cerebral white matter, basal ganglia, and dentate nucleus. The principal therapeutic strategies encompass the administration of dehydrating agents, glucocorticoids, and hyperbaric oxygen therapy. Patients with mild poisoning can achieve recovery, whereas those with severe poisoning may experience fatal outcomes. Consequently, effective preventative measures must be instituted to minimize exposure to 1,2-DCE in the workplace.

## Introduction

1,2-dichloroethane (1,2-DCE) is a halogenated hydrocarbon compound, appearing as a colorless, volatile, oily liquid at normal temperature and pressure. It plays a multifaceted role as a raw material in chemical synthesis, industrial solvent, adhesive, metal cleaner, and degreaser (Zhong et al., 2022). 1,2-DCE is not only extensively utilized in industrial applications but also plays a significant role in pharmacokinetic studies. As a commonly utilized organic phase solvent for drug dissolution, it forms a water/1,2-DCE interface, which serves as a crucial locus for the electrochemical behavior of drugs (Sobczak et al., 2024; Rudnicki et al., 2021). Through a detailed examination of the chemical behavior of drugs and biological compounds at this interface, researchers can elucidate their electrochemical properties, physicochemical parameters, and assess their stability and reactivity across varying pH conditions. For example, using 1,2-DCE as the organic phase solvent, researchers have investigated the electrochemical behavior of diverse drugs and biological compounds, including cefotaxime, β-blockers, and hemoglobin. These studies have enabled the determination of crucial parameters, such as diffusion coefficients, partition coefficients, and Gibbs free energies, thereby enhancing our understanding of drug behavior and interactions (Sobczak et al., 2024; Rudnicki et al., 2021; Herzog et al., 2008). Improper disposal of 1,2-DCE results in its release into the environment as a pollutant. Due to its persistent nature, 1,2-DCE can remain in groundwater and the atmosphere for extended periods, ultimately contributing to environmental contamination (Jeong et al., 2022; Liu et al., 2020).

**FIGURE 1:**
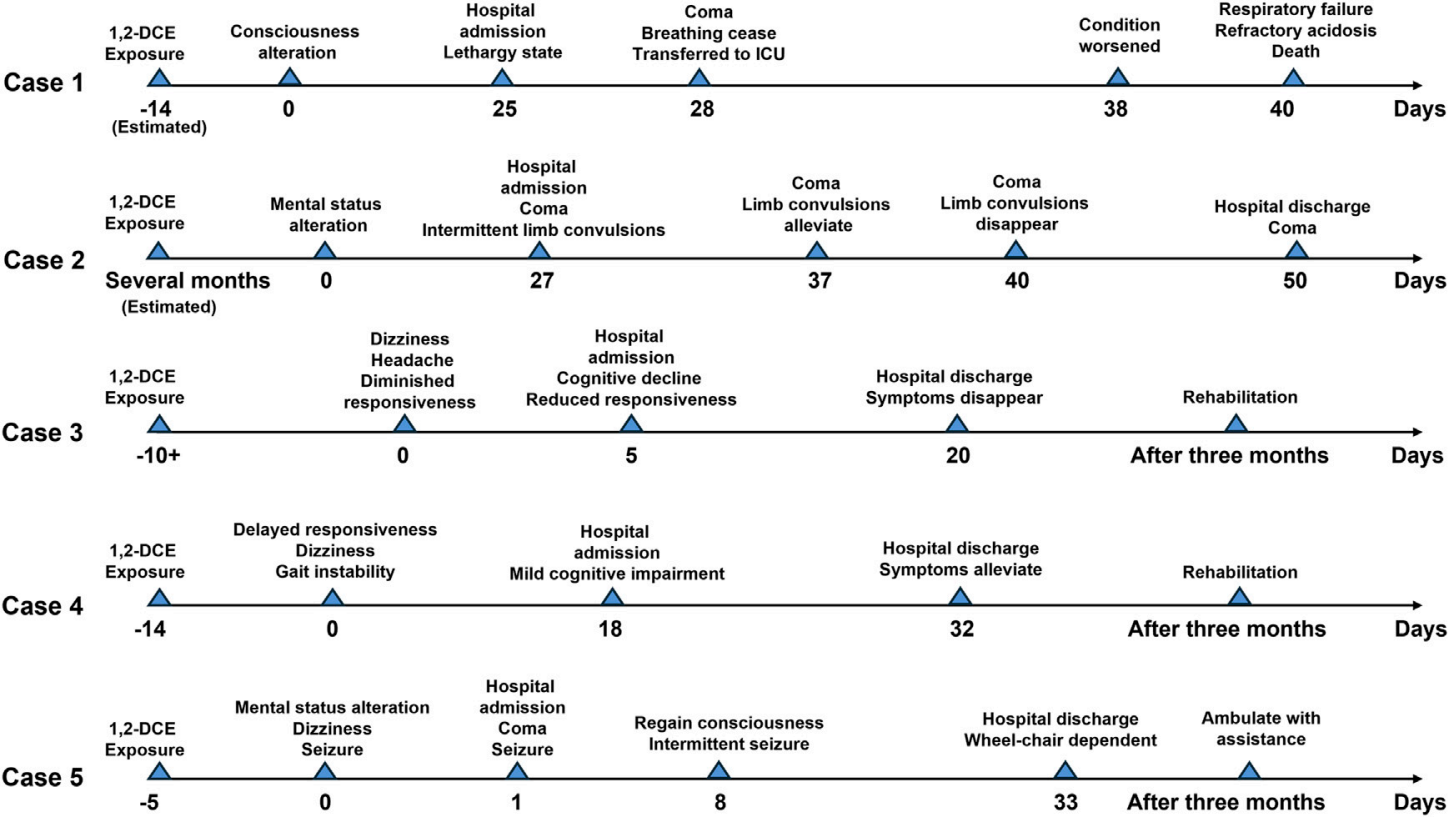
Progression timelines of the conditions for the 5 cases.

1,2-DCE possesses a distinctive chloroform-like odor and a sweet taste, and tends to evaporate during use. Notably, it is classified as highly toxic, and prolonged or inadequately protected exposure can lead to poisoning (Huang et al., 2024). There is no definitive epidemiological data on occupational exposure to 1,2-DCE. Over the past two decades, over 400 cases of acute 1,2-DCE poisoning have been reported worldwide (Xiaoyong et al., 2021). 1,2-DCE exhibits potential toxicity to the brain, liver, heart, and kidneys (Hotchkiss et al., 2010), yet its clinical manifestations are predominantly observed in the nervous system, particularly the central nervous system, manifesting as nausea, vomiting, vertigo, seizures, and coma, which are recognized as symptoms of 1,2-DCE-induced toxic encephalopathy (Liu et al., 2010). Owing to the nonspecific and frequently subtle symptoms of 1,2-DCE poisoning, misdiagnoses are prevalent. Therefore, early diagnosis and timely treatment are crucial for reducing 1,2-DCE poisoning. This article introduces five cases of 1,2-DCE poisoning, describes the clinical manifestations, treatment process, and prognosis of the patients. The progression timelines of the conditions for the five cases are illustrated in Figure 1, and a summary of the occupational history, symptoms, treatment, and outcomes for cases is presented in Table 1.

**TABLE 1:** Summary of the occupation history, symtoms and treatment of 5 cases.

Case No.	Age (yr)	Occupation	Chemicals exposed	Exposure duration	Imaging findings	Symptom	Treatment	Outcome
Case 1	31	Plumber	PVC glue	About 14 days	Hyperintensity signals in white matter and basal ganglia	Lethargic state; Decreased memory, calculation, and comprehension abilities	Mannitol; Dexamethasone; Phenobarbital; Sodium Valproate; Cefoperazone-sulbactam; Meropenem	Death
Case 2	56	Plumber	PVC glue (containing ethyl acetate and acetone)	Several months	Increased signal intensity in the bilateral frontoparietal lobes and periventricular areas	Disturbed consciousness; Recurrent limb convulsions; Comatose	Sodium valproate; Levetiracetam; Mannitol; Hyperbaric oxygen therapy; Cefoperazone-sulbactam	Coma
Case 3	37	Farmer	PVC adhesive	Over 10 days	Abnormal signal intensity in the bilateral subcortical regions, basal ganglia, bilateral thalamus, and dentate nuclei of the cerebellum	Dizziness; Headache; Diminished responsiveness	Mannitol; Methylcobalamin; Citicoline sodium	Rehabilitation
Case 4	50	Worker	PVC adhesive	14 days	Abnormal signals adjacent to the lateral ventricles and centrum semiovale	Delayed response; Dizziness; Gait instability	Mannitol, Mecobalamin; Hyperbaric oxygen therapy	Rehabilitation
Case 5	32	Worker	PVC adhesive	5 days	Abnormal signal intensity in the bilateral basal ganglia	Dizziness; Vomiting; Seizure Altered mental status	Invasive ventilation; Citicoline sodium; Hyperbaric oxygen therapy; Sodium valproic; Ceftriaxone	Ambulate with assistance

## Case reports

### Case 1

**FIGURE 2:**
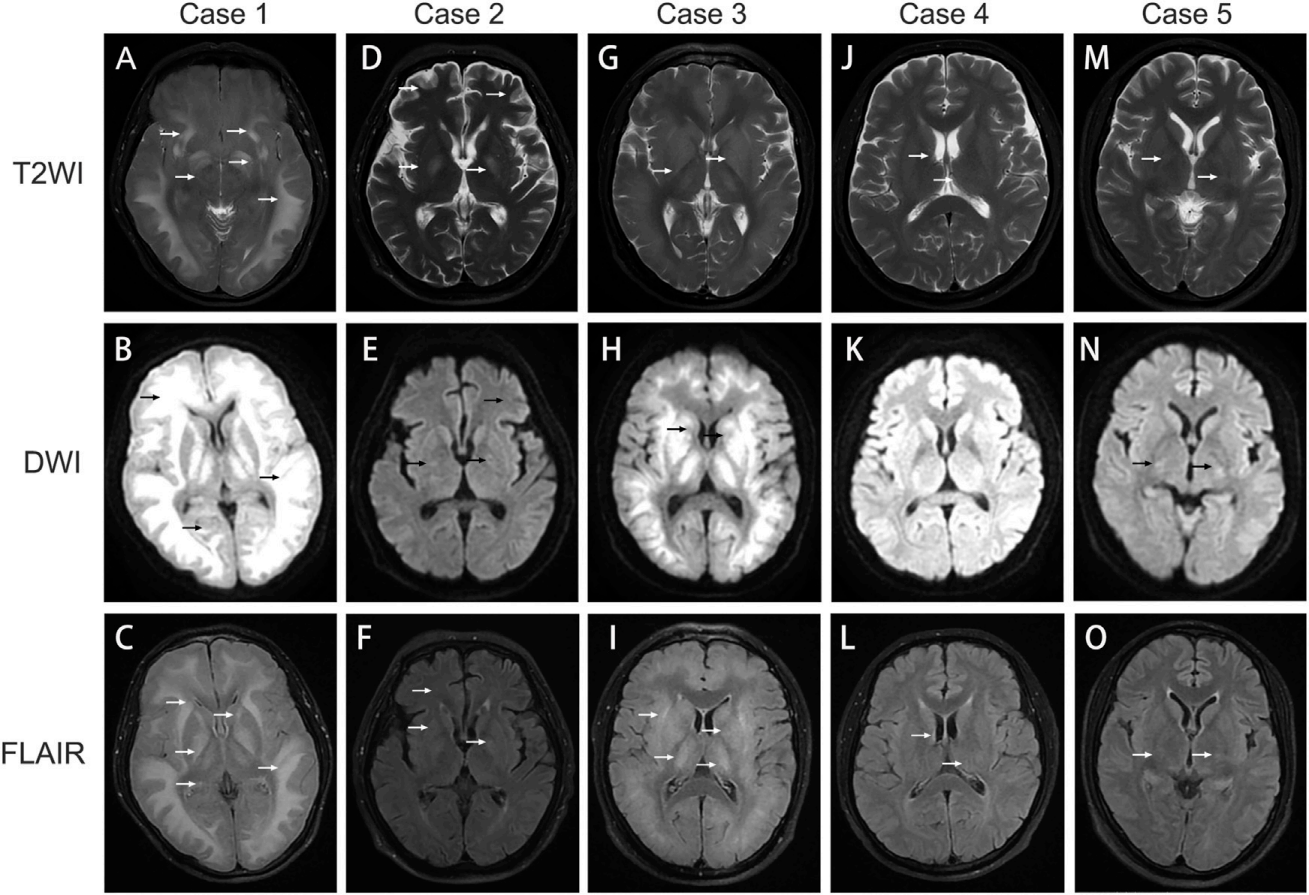
Image findings in 1,2‐DCE induced toxic encephalopathy. **(A–C)** cerebral MRI images of Case 1; **(D–F)** cerebral MRI images of Case 2; **(G–I)**: cerebral MRI images of Case 3; **(J–L)**: cerebral MRI images of Case 4; **(M–O)** cerebral MRI images of Case 5. T2WI: T2‐weighted imaging; DWI: diffusion‐weighted imaging; FLAIR: fluid‐attenuated inversion recovery. The arrows indicate cerebral lesions associated with toxic encephalopathy.

A 31-year-old male patient presented with unexplained alterations of consciousness commenced approximately 25 days prior to his clinic visit. Prior to the onset of the illness, the patient had worked as a plumber and been exposed to polyvinyl chloride (PVC) glue for approximately 14 days. During this period, he experienced intermittent recovery of consciousness accompanied by symptoms such as decreased memory, calculation, and comprehension abilities, intermittent headaches, vomiting, and diplopia. Recently, he had recurrently relapsed unconscious and was subsequently transferred to our hospital. Upon admission, the man was in a lethargic state, responsive to verbal stimuli with partial accuracy in answering questions but unable to follow commands. He exhibited decreased memory, calculation, and comprehension abilities. Vital signs remained stable, and laboratory biochemical tests as well as cerebrospinal fluid analysis yielded no significant abnormalities. Cranial magnetic resonance imaging (MRI) showed scattered hyperintensity signals in the bilateral white matter and basal ganglia on T2-weighted imaging (T2WI), diffusion weighted imaging (DWI), and fluid-attenuated inversion recovery (FLAIR) (Figures 2A–C), which indicative of toxic encephalopathy. Chest computed tomography (CT) indicated the presence of lung infection. Investigation of his occupational history revealed exposure to adhesive containing 1,2-DCE prior to the onset of illness. The man was diagnosed with 1,2-DCE induced toxic encephalopathy and received treatment with mannitol (125 mL, three times daily) for dehydration, dexamethasone (5 mg daily) to prevent cerebral edema. Levetiracetam (0.5 g, twice daily), phenobarbital, and intravenous valproate were given to alleviate seizures and convulsions. After 3 days of treatment, the patient fell into a coma, and suffered a sudden spontaneous breathing cease. Advanced life support was performed immediately, and the patient was transferred to the intensive care unit after returning spontaneous circulation. A cranial CT scan demonstrated extensive cerebral tissue swelling, with hypodensity areas in the white matter regions of the bilateral cerebral hemispheres and dentate nuclei. Treatment included mild hypothermia for neuroprotection, antiepileptic therapy with levetiracetam (0.5 g, twice daily), intravenous phenobarbital, and mannitol (125 mL, three times daily) to alleviate intracranial pressure, and cefoperazone-sulbactam (3 g, 8 h daily) for lung infection. Ten days later, the clinical condition of the man worsened, manifesting as anemia, thrombocytopenia, and coagulation abnormalities. The procalcitonin level was 12.73 ng/mL, neutrophil count was 95.9%, hemoglobin level was 61 g/L, platelet count was 9 × 10^9/L. Platelets, red blood cell suspensions, and fresh frozen plasma transfusions were administered, and meropenem (1 g, three times a day) was substituted for cefoperazone-sulbactam. During subsequent treatment, the man developed refractory acidosis and respiratory failure, ultimately resulting in death.

### Case 2

A 56-year-old male patient worked as a plumber with a history of exposure to PVC glue in a confined workspace several months ago. Approximately 27 days before admission, the patient experienced symptoms of dizziness, fatigue, vomiting, and diarrhea. Notably, two colleagues also manifested similar symptoms but recovered after changing their job assignments. A few days before hospital admission, his condition deteriorated, manifesting as disturbed consciousness with recurrent limb convulsions and incontinence. The patient visited another hospital and a brain CT scan was performed, while yielding no remarkable findings. After unsuccessful treatment, he was transferred to our hospital. Upon admission, the patient was comatose with intermittent limb convulsions, blood biochemistry tests indicated the hemoglobin level of 77 g/L, while other biochemical tests were unremarkable. Head CT scans indicated no brain parenchyma swelling, while chest CT scans revealed bilateral minimal pleural effusion and partial atelectasis with consolidation in the lower lobes of both lungs. Given the occupational exposure and symptoms of the patient, a suspicion of chemical poisoning from PVC glue arose. Detection of 1,2-DCE in the PVC glue led to a diagnosis of acute 1,2-DCE poisoning. The patient received sodium valproate (0.2 g, twice daily), and levetiracetam (0.5 g, twice daily) for seizure control, mannitol (125 mL, three times daily) to reduce intracranial pressure and alleviate cerebral edema. Hyperbaric oxygen therapy (2.0 atm of pressure, 2 hours daily) was administered to improve cerebral oxygen metabolism and promote neural function recovery. Cefoperazone-sulbactam (3 g, three times daily) was given for lung infection, and nutrition support was provided. After 10 days of hospitalization, the frequency of limb convulsions significantly diminished and completely disappeared after 13 days, but the consciousness disorders did not improve. A cerebral MRI was performed to reassess the cerebral lesions, T2WI, FLAIR, and DWI imaging disclosed multiple nodular areas of increased signal intensity in the subcortical regions of the bilateral frontoparietal lobes and periventricular areas (Figures 2D–F). Despite undergoing a 33-day treatment regimen, no significant improvement was observed in the consciousness disorders, and the man was discharged from hospital for continued rehabilitation. A 3-month follow-up showed no enhancement in his consciousness disorder.

### Case 3

A 37-year-old male patient presented with symptoms of dizziness, headache, and diminished responsiveness over 10 days before admission. He reported a history of chronic exposure to PVC adhesive in his occupational environment. Head MRI performed at a local hospital revealed symmetric abnormal high-density signals in the subcortical white matter regions of the bilateral cerebral hemispheres, brainstem, cerebellar dentate nuclei, and basal ganglia, suggesting a likelihood of toxic encephalopathy. Consequently, the patient was transferred to our hospital for further management. Upon admission, the patient demonstrated significant cognitive decline and reduced responsiveness, while other physical examinations were unremarkable. MRI findings included patchy abnormal signal intensity in the bilateral subcortical regions, basal ganglia, bilateral thalamus, and dentate nuclei of the cerebellum. The FLAIR sequence exhibited increased signal, and DWI displayed restricted diffusion, further implicating potential toxicity or metabolic encephalopathy (Figures 2G–I). The forensic identification center detected the presence of 1,2-DCE within the adhesive that the patient had been in contact with. Based on the findings, a diagnosis of acute 1,2-DCE induced toxic encephalopathy was confirmed. The man was administered mannitol (125 mL, three times daily) to alleviate intracranial pressure, along with methylcobalamin (0.5 mg intravenous daily), and citicoline sodium (0.5 g intravenous daily). Additionally, hyperbaric oxygen therapy (2.0 atm of pressure, 2 hours daily) was provided. Following more than 2 weeks of hospitalization, the symptoms disappeared. A follow-up assessment was conducted 3 months post-discharge, revealing improvement in symptoms and resumption of normal daily activities.

### Case 4

A 50-year-old man who had been working at a construction site for several months had been exposed to PVC adhesive for pipe bonding over the past 2 weeks. Over the preceding 18 days, he developed symptoms of delayed response, dizziness, and gait instability. These symptoms intensified the day before hospital admission, accompanied by involuntary tremors in all extremities. Upon admission, physical examination disclosed decreased reactivity and mild cognitive impairment, particularly in calculation and orientation skills. Initial laboratory tests did not reveal any abnormalities. A cranial MRI demonstrated striped and patchy abnormal signals adjacent to the lateral ventricles and centrum semiovale in T2WI and FLAIR, while no significant abnormal signals were detected on DWI (Figures 2J–L). The electromyogram of the limbs indicated a slight decrease in the sensory wave amplitude of the left ulnar nerve compared to the right, with normal conduction velocity. The F-waves of bilateral ulnar, median, and tibial nerves were normal, and the H-reflexes of bilateral tibial nerves were within normal ranges. The man received treatment with mannitol (125 mL, three times daily), mecobalamin, and hyperbaric oxygen therapy (2.0 atm of pressure, 2 hours daily). Following approximately 2 weeks of treatment, his reactivity enhanced, dizziness diminished, and extremity tremors resolved. At a 3-month follow-up appointment after discharge, the cognitive function, calculation abilities, and orientation had notably improved, without any adverse effect on his daily activities.

### Case 5

A 32-year-old man was admitted to our hospital with a 1-day history of dizziness and vomiting, followed by a prolonged seizure episode lasting over 10 h, accompanied by altered mental status. The patient had been exposed to PVC adhesive in a poorly ventilated, approximately 1-square-meter with a height of 3-m workplace while brushing pipes. The man did not wear protective gloves during work and only wore a regular protective face mask. He worked continuously for 6 days before hospital admission, approximately 5–7 h each day. On the preceding day, the patient experienced dizziness, nausea, vomiting, and developed to coma. Upon presentation, he exhibited tachypnea and seizures involving all four limbs. Laboratory tests revealed the interleukin-6 was 69.45 pg/mL, and procalcitonin was 0.75 ng/mL, and the lactate level was 5.67 mmol/L. Elevation in white blood cell count, alanine aminotransferase and aspartate aminotransferase was revealed. Cranial MRI demonstrated abnormal signal intensity in the bilateral basal ganglia (Figures 2M–O), while no abnormality was detected on head CT. Chest CT imaging demonstrated bilateral lung infections with consolidation in the lower lobes and minimal pleural effusion. The patient received invasive ventilation, citicoline sodium (0.5 g intravenous daily), along with hyperbaric oxygen therapy (2.0 atm of pressure) twice-daily. Sodium valproate and ceftriaxone (2 g daily) were administered intravenously for seizure management and pulmonary infection. After 7 days of treatment, the patient regained consciousness and was successfully weaned off ventilator support, although intermittent seizures and restlessness persisted. After 32 days of hospitalization, the motor function of the man exhibited improvement, however, he remained non-ambulatory and wheelchair-dependent. Consequently, the patient was discharged for rehabilitation therapy. During the 3-month follow-up, the man demonstrated the ability to ambulate with assistance.

## Discussion

We provided a case series related to occupational toxic encephalopathy caused by 1,2-DCE exposure, with detailed occupation history, symptoms, neuroimaging findings, treatment, and outcomes. This case series contributes valuable insights to the field of occupational toxicology and neurology.

Occupational exposure to 1,2-DCE is acknowledged as the primary cause of 1,2-DCE poisoning, with dermal and inhalation routes being the principal pathways of exposure. Inadequate use of protective equipment and working in poorly ventilated environment increase the risk of occupational poisoning (Nouchi et al., 1984). Upon inhalation or dermal exposure, 1,2-DCE is absorbed into the body via the lungs and distributed throughout various tissues through bloodstream, then distributes to various organs, including the lungs, brain, liver, kidneys, blood, and adipose tissues (Take et al., 2013). Due to its high lipid solubility, 1,2-DCE significantly accumulates in abdominal fat, and brain tissue (Reitz et al., 1982; Kwak et al., 2018). The mechanism of 1,2-DCE induced neurotoxicity remains incompletely elucidated. Current researches have revealed that reactive oxygen speciesplay a crucial role in the formation of brain edema, while the Ca^2+^ overload and disruption of the blood-brain barrier can lead to brain edema and inflammatory responses, thereby influencing the pathogenesis of brain edema. Furthermore, 1,2-DCE can perturb neurotransmitter changes, disrupt energy metabolism, and modulate the expression of aquaporin 4 (Xiang et al., 2023). Excessive exposure to 1,2-DCE results in elevated serum levels of aspartate aminotransferase and alanine aminotransferase. It also leads to oxidative stress-mediated accumulation of hepatic glycogen, free fatty acids, and triglycerides in mice, consequently disrupting lipid homeostasis (Xiang et al., 2023; Wang et al., 2017). 1,2-DCE exposure causes proximal tubular nephropathy and impair the regeneration of renal tubular epithelial cells in rats. Chloroacetaldehyde is a metabolic product derived from the biotransformation of 1,2-DCE. Chloroacetaldehyde has been shown to exert nephrotoxic effects through inhibition of oxidative phosphorylation and a reduction in adenosine triphosphate production (Knouzy et al., 2010). In an *in vitro* experimental model using isolated rat atria, cardiovascular toxicity induced by chloroacetaldehyde was observed, characterized by calcium channel-mediated myocardial tension inhibition (Chen et al., 2011). Moreover, 1,2-DCE can induce sperm malformation and contribute to the development of malignant tumors (Xiang et al., 2023).

Previous reports indicated that neurological damage caused by short-term exposure to 1,2-DCE was generally reversible; however, long-term exposure often led to severe and irreversible neurological impairment (Kwak et al., 2018; Chen et al., 2015). Consistent with these findings, patients in case 3, 4 and 5 experienced approximately complete resolution of neurological symptoms after treatment, Conversely, the patient rin case 1 suffered from progressively worsening cerebral edema induced by 1,2-DCE, leading to respiratory and cardiac arrest, and the patient in case 2 remained in a persistent coma following 1,2-DCE poisoning, without regaining consciousness. Other case reports have reported the onset of 1,2-DCE poisoning symptoms within weeks to months of exposure (Dang et al., 2019). In this case report, we observed that 1,2-DCE poisoning may manifest neurological damage within 1 week to approximately 1 month post-exposure, with a latent onset characterized by initial neurological symptoms. The mild clinical presentations initially include dizziness, headache, nausea, and vomiting, accompanied by decreased responsiveness and impairments in calculation, memory, and cognitive function. Prolonged exposure to 1,2-DCE can exacerbate poisoning, which leads to increased intracranial pressure, limb convulsions, and coma. Cerebral edema is a prominent feature of 1,2-DCE poisoning, which can persist from 2 weeks to beyond a month. Another notable characteristic is the sudden exacerbation of cerebral edema after initial alleviation, which may result in abrupt condition deterioration, irreversible central nervous system damage, and cardiac arrest due to sudden intracranial pressure elevation. In addition, coma caused by 1,2-DCE poisoning can impair airway protection and increase the risk of aspiration pneumonia.

1,2-DCE poisoning often has an insidious onset and suddenly deteriorates. Prolonged cerebral edema is a significant clinical feature of 1,2-DCE poisoning (Dang et al., 2019). Head CT scans frequently fail to detect lesions in the early stages, whereas cranial MRI plays a crucial role in diagnosing 1,2-DCE-induced toxic encephalopathy. Head MRI reveals symmetric, diffuse abnormal signal intensity in the bilateral subcortical white matter and basal ganglia area, particularly affecting the cerebellar dentate nucleus, manifesting as high-intensity signals on T2WI and DWI (Zhan et al., 2011; Liu et al., 2019). Other studies have indicated that brain lesions can be observed in regions such as the centrum semiovale, external and internal capsule, thalamus, and even the brainstem and cerebellar hemisphere during the acute phase. In this phase, brain edema primarily arises from cytotoxic mechanisms, while in the subacute phase, the edema mainly attributed to vasogenic factors (Liu et al., 2010; Ridgway et al., 2003). DWI has the capability to detect brain alterations at an earlier stage compared to conventional T2WI (Chen et al., 2015). Notably, a thorough investigation of the occupational exposure history is crucial to prevent misdiagnosis. Intracranial infections or metabolic disturbances can also lead to coma and seizures. Laboratory examinations including cerebrospinal fluid analysis and blood gas analysis, as well as comprehensive physical examinations were necessary for differentiating these conditions.

There is no specific antidote for 1,2-DCE poisoning. In the early stages, glucocorticoids, hyperosmolar diuretics such as mannitol, and dehydrating diuretics like furosemide are useful to alleviate cerebral edema. Dehydrating agents should be administered in adequate amounts for a full course, with a gradual reduction. Anticonvulsants like sodium valproate, levetiracetam, and hyperbaric oxygen therapy to enhance cerebral oxygen metabolism are recommended to facilitate neurological recovery. In the report by Cai et al., hyperbaric oxygen therapy was also administered to treat toxic encephalopathy resulting from 1,2-DCE exposure (Cai et al., 2024). Nevertheless, reports on the use of hyperbaric oxygen for DCE poisoning are quite scarce. Our case report presents the viability of utilizing hyperbaric oxygen therapy for 1,2-DCE induced encephalopathy. Further studies are warranted to explore the therapeutic efficacy of hyperbaric oxygen therapy in managing 1,2-DCE induced encephalopathy. The irrational use of 1,2-DCE in industrial products is prevalent in developing countries, posing a threat to the health of workers. Therefore, it is imperative to enhance the provision of personal protective equipment and intensify occupational health training.

This case series has the following limitations. Firstly, since 1,2-DCE can enter the body through inhalation or skin exposure, accurately quantifying the precise concentration and dosage of 1,2-DCE in the PVC glue to which the patients were exposed in their occupational environments is difficult. The assessment of 1,2-DCE exposure primarily relies on patients’ occupational histories and self-reports, which may be subject to recall bias or inaccuracies in the information provided. Secondly, the small sample size in this case report limits its ability to comprehensively represent the characteristics and prognoses of all patients with 1,2-DCE poisoning. Lastly, the absence of longer-term follow-up for the patients restricts our ability to conduct a thorough evaluation of the chronic effects of 1,2-DCE-induced toxic encephalopathy, as well as its potential impacts on other organs.

## Conclusion

1,2-DCE is a commonly used compound in diverse industrial products. Prolonged exposure to 1,2-DCE without adequate occupational protection poses the risk of acute poisoning. The diagnosis of 1,2-DCE-induced encephalopathy is contingent upon a thorough evaluation encompassing exposure history, clinical presentation of encephalopathic symptoms, and neuroimaging abnormalities. Notably, hyperintensity signals in the subcortical white matter and basal ganglia on T2WI and DWI of cranial MRI are important characteristics in the early diagnosis of 1,2-DCE-induced encephalopathy. Cerebral edema, a potentially life-threatening complication associated with 1,2-DCE poisoning, necessitates prompt therapeutic interventions. Implementing rigorous protective measures in the workplace is necessary to minimize 1,2-DCE exposure and decrease the risk of poisoning.

## Data Availability

The original contributions presented in the study are included in the article/supplementary material, further inquiries can be directed to the corresponding author.
